# An Experiment-Based Study of Formation Damage Using a Microetching Model Displacement Method

**DOI:** 10.3390/mi13020270

**Published:** 2022-02-08

**Authors:** Feng Wu, Jin Dai, Lei Shi, Lin Fan, Yao Guan, Yuhan Li, Qinghui Wang, Chunchao Chen

**Affiliations:** 1 School of Geoscience and Technology, Southwest Petroleum University, Chengdu 610500, China; hibiscidai@163.com (J.D.); chunyilao@126.com (C.C.); 2 Research Institute of Shenzhen Branch, CNOOC China Limited, Shenzhen 518067, China; shilei22@cnooc.com.cn (L.S.); guanyao@cnooc.com.cn (Y.G.); wangqh24@cnooc.com.cn (Q.W.); 3 Middle Sichuan Oil and Gas Field, PetroChina Southwest Oil & Gas Field Company, Suining 629000, China; flcz@petrochina.com.cn (L.F.); lisongbai01@petrochina.com.cn (Y.L.)

**Keywords:** laser, etching, microscopic model, drilling fluid, formation damage

## Abstract

In the field of oil and gas exploration, drilling fluid is regarded as the essential “blood” for drilling, which mainly helps to control the formation pressure and remove cuttings from the well. During the drilling fluid cycle, the drilling fluid penetrates into the pores of the formation rock, thus blocking the rock pores and resulting in a decline in oil and gas recovery efficiency. Therefore, it is very important to understand the microscopic mechanism of formation damage caused by drilling fluid. However, as an important component of formation damage, the microscopic mechanism of fluid damage has not yet been clearly revealed. In this study, a new microetching model (MEM), along with displacement equipment, was designed. The pore network of rock samples was extracted from thin-section images and etched to a thin aluminum sheet by laser. Oil-based drilling fluid was used to displace the stratum water in the MEM. The displacement process was recorded by a camera and analyzed. A core flooding experiment, permeability measurement, and SEM observations were performed. The results show that, for low-porosity and low-permeability sandstone, the main forms of formation damage by drilling fluid include solid damage and liquid damage. Solid damage is mainly caused by the blockage of small pores and narrow throats with solid particles of the size 0.1~30.0 μm in drilling fluid, while liquid damage is mainly caused by the water lock and hydrocarbon lock effects formed by the oil–water two-phase interface, gas–water two-phase interface, or the oil–gas–water three-phase interface.

## 1. Introduction

**Figure 1:**
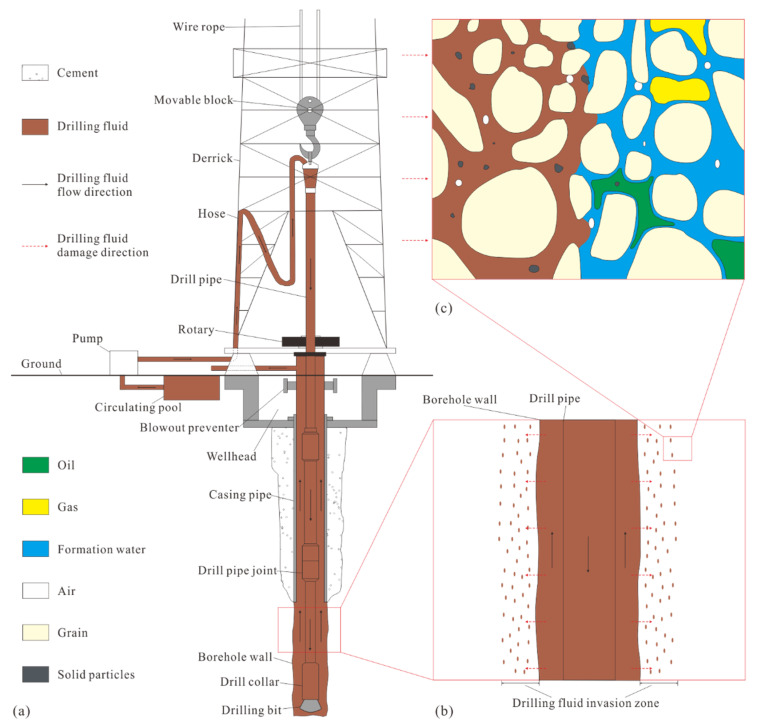
Schematic diagram of drilling fluid cycle and formation damage: (**a**) diagram of drilling fluid cycle during well drilling; (**b**) diagram of drilling fluid invading formation and causing formation damage; (**c**) diagram of microscopic formation damage.

In drilling for oil and gas exploration, the drilling fluid in the wellbore mainly plays the role of controlling the formation pressure and removing cuttings from the well [1,2,3,4] (Figure 1a). During the drilling fluid cycle, the drilling fluid infiltrates the formation rock through pores, thus blocking the pores in rock (Figure 1b,c) and reducing the permeability of the formation, resulting in a dramatic decline in oil and gas recovery efficiency [5,6,7]. In low-porosity and low-permeability sandstone reservoirs [8,9,10,11,12], the formation damage from drilling fluid is extremely complicated. Therefore, with the gradual advancement of exploration and the development of low-porosity and low-permeability sandstone reservoirs, the study of the microscopic mechanism of formation damage by drilling fluid has received increasing attention in recent years.

At present, the experimental methods for studying the damage to rocks caused by drilling fluid at the microscopic scale include thin-section observations [13,14], scanning electron microscope (SEM) observations [15,16], X-ray diffraction (XRD) [17], nuclear magnetic resonance (NMR) imaging [18], CT scanning [19], etc. The main idea of these methods is similar: first, obtaining rock samples (from drilling) before drilling fluid damage and rock samples (from core flooding experiments) after drilling fluid damage and then scanning or observing the rock samples before and after drilling fluid damage at the microscopic scale, and comparing the differences between them to determine the characteristics of formation damage. Among these methods, the advantage of both the thin-section and SEM observation methods is that high-resolution microscopic images can be obtained, which can characterize the formation damage caused by the solid particles from drilling fluid blocking the pores [13,14,15,16]. The advantage of the XRD method is that the influence of the composition and content of the solid particles in drilling fluid on the formation damage can be quantitatively analyzed [17]. The advantage of the NMR imaging and CT scanning methods is that the damage of core samples by drilling fluid can be observed from a three-dimensional scale, but the resolution of the images is relatively low, such that the characterization of the microscopic details is insufficient [18,19,20]. In addition, some studies have shown that drilling fluids with different liquid components (oil and water) undergo changes in viscosity, dynamic shearing force, static shearing force, wettability, and other properties under formation temperature and pressure conditions, which lead to complex formation damage characteristics [21,22]. Thus, the liquid components of drilling fluid play important roles in formation damage. However, these existing methods also have a common drawback: they cannot characterize the formation damage caused by liquid components in drilling fluids.

In recent years, with the continuous progress of photolithography, the accuracy of etching is improving, and the field of its application is expanding [23,24,25]. Some studies utilized photolithography to create microetching models (MEMs), which were then used for seepage simulation of oil, gas, and water in rock pores, which achieved good results [26,27,28,29,30,31,32]. In these microscopic models, a glass sheet is etched, leaving micronscale grooves on the surface of the glass sheet (these grooves replicate the pore structure of the rock), and then, the etched glass sheet is covered with another piece of glass. By laminating the two together with pressure, a fluid displacement experiment can be conducted in the grooves, which represent the pore structure. The advantage of this technique is that the flow characteristics of the fluid in the pore structure can be observed visually and clearly. However, since the lamination of the glass sheets is maintained only by applying unbalanced pressure around the edges of the model, only a relatively thin glass sheet can be used, so the microscopic model can withstand only a very limited pressure, and the fluid displacement pressure used in the experiment can only be much lower than the pressure difference under the formation conditions, otherwise, the microscopic model will be damaged due to uneven force. Since the viscosity of drilling fluid is much higher than that of light crude oil, natural gas, and stratum water, and the actual pressure difference in the well during the drilling process is also higher, this technique cannot be used to perform microscopic simulation experiments of formation damage by drilling fluid.

The aim of this study is to design a new MEM and fluid displacement technique that involves replicating the pore structure of rock on aluminum sheets using infrared lasers and encapsulating the models with thicker glass and rubber to create MEM that can withstand higher pressures, so as to continue the drilling fluid displacement of stratum fluids in rock pore structures. This new technique can reveal the microscopic mechanism of formation damage caused by liquid components and provide a theoretical basis for the efficient exploration and development of oil and gas.

## 2. Experiments

### 2.1. Micromodel Fabrication

**Figure 2:**
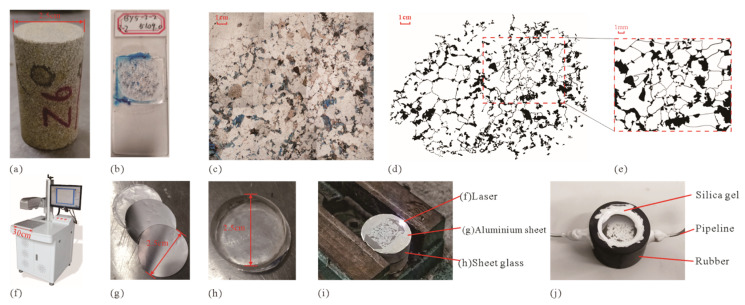
Microetching modeling fabrication process: (**a**) core plug; (**b**) casting thin section; (**c**) microscopic image of the casting thin section; (**d**) rock core throat structure extraction; (**e**) microscopic model core throat structure; (**f**) industrial laser marking machine; (**g**) thin aluminum sheet; (**h**) transparent thick glass sheet; (**i**) laser engraving; (**j**) MEM after encapsulation.

The first step of MEM fabrication is to extract the pore structure from the rock (Figure 2a–e). A standard plug sample (diameter 2.5 cm, length 3.0–10.0 cm, Figure 2a) was drilled from the downhole core, from which a small section was intercepted. Liquid dye resin (usually blue or red) was injected into the rock pore under pressure in a vacuum, and when the liquid dye resin cools and solidified, the rock sample was ground into a 0.03 mm thick mineral sheet and encapsulated with a 1.0 mm thick glass carrier sheet and a 0.1–0.2 mm thick glass cover sheet to obtain the rock casting thin section (Figure 2b). The casting thin section was observed using an optical microscope, and microscopic photographs of the rocks were taken, in which the pores were filled with dye resin in blue or red, while the rock particles remained white or gray. By using image processing techniques (threshold method [32,33], watershed method [34], edge extraction method [35], etc.), the rock pore structure was extracted based on the microscopic images (the black part represents the pore network, and the white part represents the rock grain; Figure 2d). The representative area from the binarized pore structure image was extracted as the pore structure of the microscopic model (Figure 2e).

The second step of MEM fabrication is to etch the pore structure (Figure 2f–i). Previous studies used the photochemical etching method to process the thin glass sheet, so the pressure that the microscopic model can withstand is minor [36,37,38,39]. In this study, laser etching of the thin aluminum sheet was used. An RJ100W fiber laser marking machine, produced by Chongqing Renbao Technology CO., LTD. (Chongqing, China), was used in this research (Figure 2f). The laser marking machine emits a high-energy laser beam from its top optical cylinder to burn the object and, thus, penetrate it or form a groove on its surface, with a laser printing range of 110 mm × 110 mm and a single-beam laser resolution of 1 μm. The thin aluminum sheet (40 μm in thickness) with good thermal conductivity was selected for this study so that it could be penetrated by the laser. With the pore structure (Figure 2e) as a model, the thin aluminum sheet was engraved by the laser, and the part of the aluminum sheet that burned off represents the pores, as well as the fluid inlet and a fluid outlet, while the remaining part represents the rock grain (Figure 2i).

The third step of MEM fabrication is encapsulation (Figure 2j). To facilitate the observation of the seepage characteristics of the microscopic model, the microscopic model engraved by the aluminum sheet was sandwiched between two transparent thick glass sheets (2.5 cm in diameter and 1 cm in thickness), and then, the two glass sheets with the microscopic model were wrapped with a rubber sleeve, and the contact between the top and bottom of the rubber sleeve and the glass sheet was sealed with silicone. Two openings were made on opposite sides of the rubber sleeve, and then, two hollow metal pipelines (3 mm of outer diameter and 2 mm of inner diameter) passed through the openings and were connected to the fluid inlet and outlet of the microscopic model, respectively. The contact between the metal pipelines and the rubber sleeve was also sealed with silicone (Figure 2j). After applying fluid pressure around the encapsulated model, the displacement fluid could only flow through the hollow metal pipelines to the burned part of the aluminum sheet (representing the pores in rock).

### 2.2. Drilling Fluid Displacement

**Figure 3:**
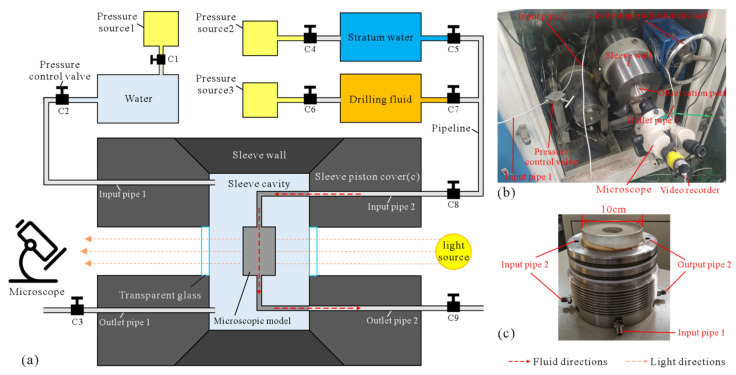
Experimental device: (**a**) schematic diagram of the cross section of the microdisplacement experimental device; (**b**) image of the experimental device assembly; (**c**) stainless steel sleeve cavity.

After obtaining the MEM, the drilling fluid displacement of stratum fluids was carried out through the microetching displacement device, which simulates the damage of the reservoir by drilling fluid under drilling conditions. In this study, the major part of the MEM displacement device (Figure 3a,b) was a sleeve cavity made of 304 stainless steel (Figure 3c), and a window made of transparent glass (3 cm in thickness) was reserved in the center of the sleeve cavity. The connection between the transparent glass and the sleeve cavity was sealed with a waterproof adhesive, which ensures the pressure inside the sleeve cavity and also facilitates the real-time observation of the experimental process. After the encapsulated MEM is placed into the sleeve cavity, the input pipe of the encapsulated MEM was connected to the container for storing stratum water and drilling fluid. The displacement pressure was applied to the stratum water and drilling fluid through pressure devices, and the fluid outlet was connected to the outlet pipe, with control valves in all pipes. The input pipe of the annulus between the sleeve cavity and the encapsulated MEM was connected to a container for pure water, and the confining pressure was applied to the encapsulated MEM by a pressure device, while the outlet of the annulus was connected to the outlet pipe, with control valves in all pipes. A light source was provided on one side of the observation window, and a microscope and video recorder were set up on the other side.

The stratum water used for the microetching model displacement experiments was blended according to the ion concentrations of the actual stratum water, and methyl blue was added to facilitate differentiation from the pure water (transparent) in the annulus between the sleeve cavity and the encapsulated MEM. The drilling fluid used in this research was oil-based drilling fluid. The composition of the oil-based drilling fluid is listed in Table 1. The pressure source is a 10 MPa nitrogen cylinder or an electric oil pump. During the experiment, the annulus between the sleeve cavity and the encapsulated MEM was filled with pure water, and the confining pressure (generally 3 MPa) was applied to the microscopic model by the pure water to make sure that the two pieces of thick glass in the microscopic displacement model clamp the aluminum sheet, thus ensuring that the drilling fluid displacement pressure (generally 1.0–1.5 MPa) would not damage the microscopic model. If the confining pressure and displacement pressure required by the experiment are relatively high, the confining pressure and displacement pressure should alternately be gradually increased, and the confining pressure should always be kept higher than the displacement pressure.

The main process of the microscopic displacement experiment is as follows: (1) Experimental device assembly: The displacement experimental device was assembled in accordance with Figure 3a, and the completed assembly of the displacement experimental device is shown in Figure 3b. The sealing state of each interface of the instrument was checked, and each valve was closed. The light source was turned on, and the focal length of the microscope was adjusted to ensure clear observation of the microscopic model. (2) The sleeve cavity was filled with pure water to provide confining pressure: Valves C2 and C3 were opened, followed by valve C1, and pure water was injected into the annulus until the C3 mouth had a stable outflow of pure water. At this time, the annulus completed water injection, and valves C3, C2, and C1 were shut in order. (3) Microscopic model saturated with stratum water: Valves C9, C8, and C5 were opened, followed by valve C4, and the pressure was adjusted so that the stratum water slowly flowed into the microscopic model. At the same time, a microscope was used to observe and record the displacement process. Until the stratum water flowed out steadily at valve C9, there was almost no gas in the pores of the microscopic model, as observed by the microscope, which means the model was saturated with stratum water. Then, valves C4, C5, and C8 were closed. (4) Drilling fluid displacement of stratum water: Valves C7 and C8 were opened, and then, valve C6 was slowly opened, and the pressure was controlled so that the drilling fluid slowly entered the water-saturated microscopic model while observing with the microscope and filming the displacement process with the camera. The displacement pressure was maintained until there was no fluid flowing out at valve C9 for a period of time (e.g., 60 min), at which time there was no more fluid flowing in the pores of the microscopic model, as observed by the microscope, representing the maximum degree of the damage of the microscopic model by drilling fluid. (5) The pressure was released, all valves were closed, the device was disassembled, and the experiment ended.

### 2.3. Permeability Measurement and SEM Observations

To further verify the degree of the damage to cores caused by drilling fluid, four core plugs were also selected to carry out flooding experiments on the core plugs by drilling fluid, and the permeability of the core plugs before and after the damage was measured for comparison. The permeability measurement was performed with a Core Lab CMS^TM^-300 automated permeameter using the unsteady-state method following the standard SY/T 6385-2016. The fluid used for permeability measurement was nitrogen at a temperature of 20 °C, a confining pressure of 5 MPa, and an initial displacement pressure of 3 MPa. When the gas flow rate reaches a stable state, the flow rate and pressure value are read, the permeability can be obtained by Equation (1) [40]. The permeability decline ratio of the rock sample caused by drilling fluid damage can be obtained by Equation (2).

(1) $$K=\frac{2\;p_{a}\;Q\;\mu \;L}{A\;\left(p_{1}^{2}-p_{2}^{2}\right)}\times 10^{3}$$

where *K* is the permeability (mD); *A* is the cross-sectional area of the rock sample (cm^2^); *L* is the length of the rock sample (cm); *p*_1_ is the inlet pressure (MPa); *p*_2_ is the outlet pressure (MPa); *p_a_* is the atmospheric pressure (MPa); *Q* is the flow rate (cm^3^/s); *μ* is the test gas viscosity (MPa·s); nitrogen has a fluid viscosity of approximately 0.0173 MPa·s at a temperature of 20 °C and one atmosphere.

(2) $$R_{d}=\frac{K_{1}}{K_{2}}\times 10^{2}$$

where *R_d_* is the permeability decline ratio (%); *K*_1_ is the permeability of the rock sample before the damage (mD); *K*_2_ is the permeability of the rock sample after damage (mD).

The core plugs before and after damage were cracked open to obtain their natural sections, and SEM observations were used to reveal the blockage of rock pores by solid particles from the drilling fluid. The SEM observation was performed by an FEI Quanta 650 FEG scanning electron microscope.

## 3. Geological Setting and Samples

The rock samples used in this study were collected from the Enping Formation in the Baiyun Sag of Pearl River Mouth Basin [41]. During the deposition of the Enping Formation, the area mainly developed super large deltas [42]. Previous drilling wells show that the delta has high sand content; the distributary channel is well developed; the particle size of the rock is coarse, and the lithology is dominated by coarse sandstone [43]. The pore structure of the reservoir rock is complex, with the porosity mainly distributed between 6% and 15%, and the permeability mainly distributed between 0.45 mD and 8.8 mD. The reservoir is characterized by low porosity and low permeability [44]. The rock samples used in this study were drilled from the stratum with a depth of 3734–3769 m. Six core plugs were used to make casting thin sections, and the microscopic pore structures were obtained, and MEMs were created. Another four supporting core plugs were used to carry out the core flooding experiment by drilling fluid and subsequent permeability measurement and SEM observations.

## 4. Results and Discussion

**Figure 4:**
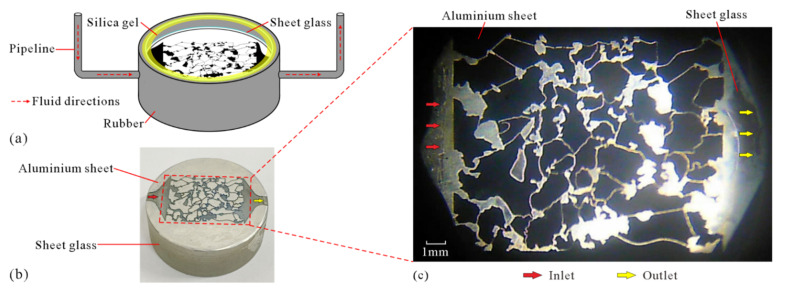
MEM and photos taken under microscope: (**a**) schematic diagram of MEM after encapsulation; (**b**) MEM attached to a single piece of glass; (**c**) image of MEM under microscope.

Figure 4a is a schematic diagram of the MEM after encapsulation, and the MEM encapsulated in the rubber sleeve is shown in Figure 4b. In Figure 4b, inside the red dotted rectangle, the area burned off by the laser and showing the bottom glass sheet represents the pores. Outside the red dotted rectangle, the area burned off by the laser and showing the bottom glass sheet represents the inlet and outlet of the fluid (indicated by the red and blue arrows, respectively). In the microscopic displacement experimental device, when the MEM is assembled and the fluid has not yet been injected, the light passes through the pores and the inlet and outlet parts of the MEM and can be photographed on the microscopic side of the observation window, as shown in Figure 4c. At this time, the light-transmitting white areas represent the pores and fluid inlets and outlets, while the opaque black areas represent the rock grain.

**Figure 5:**
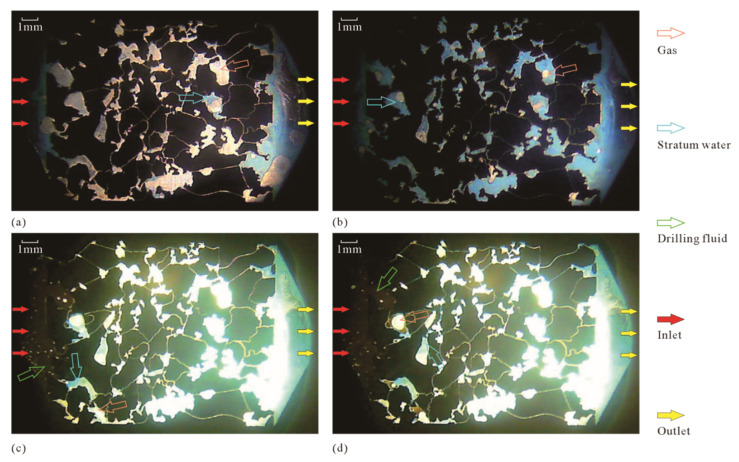
Experimental image of the damage of MEM by drilling fluid: (**a**) intermediate state of saturated stratum water in the microscopic model; (**b**) final state of saturated stratum water in the microscopic model; (**c**) intermediate state of drilling fluid displacing stratum fluid; (**d**) completed state of drilling fluid displacing stratum fluid.

Figure 5a,b, respectively, show the intermediate state and the final state of the microscopic model saturated with water. During the saturation process of the microscopic model, dyed stratum water (blue) gradually appears in the pores (white), which were filled with gas in the initial state, indicating that the stratum water gradually displaces the gas in the pores (Figure 5a). In the final state in which the microscopic model is saturated with water, the pore throat of the microscopic model is almost completely filled with blue stratum water, but a small amount of gas remains in some poorly connected pores (Figure 5b). Figure 5c,d, respectively, show the intermediate state and the completed state of drilling fluid displacement. The drilling fluid used in this study was oil-based drilling fluid and, therefore, was brown under the microscope. In the initial stage of drilling fluid displacement, the drilling fluid flows faster and quickly reaches the entrance of the microscopic model (Figure 5c). As the drilling fluid displacement progresses, the speed of the drilling fluid slows down significantly. When the confining pressure and displacement pressure are kept stable for more than 20 min, the distribution of each fluid no longer changes significantly, and the completion state of the drilling fluid displacement is obtained (Figure 5d).

**Figure 6:**
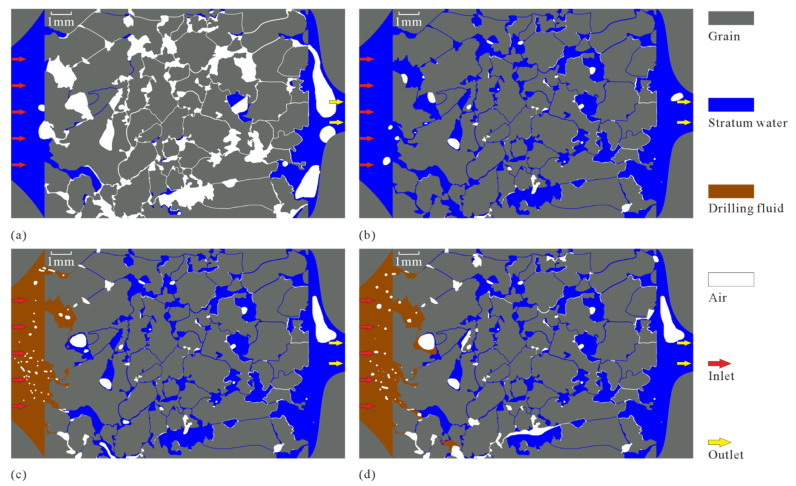
Image processing results of the damage of MEM by drilling fluid: (**a**) intermediate state of saturated stratum water in the microscopic model; (**b**) final state of saturated stratum water in the microscopic model; (**c**) intermediate state of drilling fluid displacing stratum fluid; (**d**) complete state of drilling fluid displacing stratum fluid.

In order to observe the distribution state and contact relationship of drilling fluid, stratum water, and gas in the MEM more clearly, and to quantitatively analyze the volume change in different fluids during the displacement, image processing was performed on the photos taken by optical microscope (Figure 6). The processed images in Figure 6a–d correspond to the initial images of Figure 5a–d, respectively. In Figure 6, the gray color represents the rock grain (aluminum sheet), the blue color represents the stratum water, the white color represents the gas, and the orange color represents the drilling fluid.

The image of the microscopic model saturated with water after image processing (Figure 6a) clearly shows that the stratum water first rapidly reaches the outlet end from the inlet end through the dominant channels (connected large pores and throats) and then gradually displaces the gas from the pores and throats. During the saturation of the stratum water in the microscopic model, some of the gas in the pores is not completely removed (this phenomenon is similar to the case of some of the pores being occupied by oil or gas in the reservoir). The remaining gas is mainly in the disconnected pores and small throats, the connection between large pores and throats, etc. (Figure 6b).

The image of the microscopic model of drilling fluid displacing stratum fluid after image processing (Figure 6a) clearly shows that, in the process of the damage of the model by drilling fluid, the oil from drilling fluid, stratum water, and gas are mixed with each other, forming a large number of bubbles, water droplets and oil droplets, which results in a large number of two-phase fluid or even three-phase fluid interfaces, with intense surface tension. When these interfaces move to narrow pores and throats, a very large resistance is generated, which makes fluid percolation extremely difficult, forming water and hydrocarbon lock effects [45,46].

**Figure 7:**
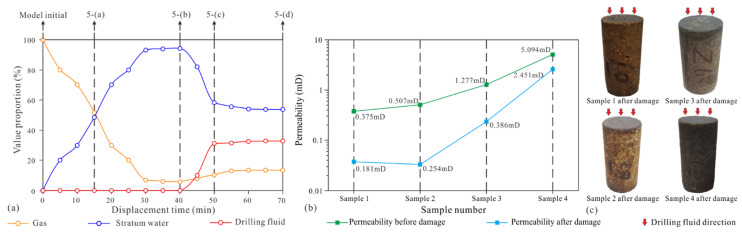
Quantitative analysis of formation damage by drilling fluid: (**a**) change in drilling fluid, gas, and water ratios with time for the microscopic model; (**b**) comparison of permeability of rock samples before and after damage by drilling fluid; (**c**) photos of rock samples after damage by drilling fluid through core flooding experiments.

More images during the microscopic model saturated with stratum water and drilling fluid displacement experiments are selected for image processing, and the volume percentages of different fluids (stratum water, gas, and drilling fluid) are calculated and plotted as a line graph of their change with displacement time (Figure 7a). In the microscopic displacement experiment with a duration of 70 min, the microscopic model saturated with stratum water ranges from 0 to 40 min. From 0 to 30 min, the stratum water fills the pores of the microscopic model at a nearly uniform rate and approaches the saturation of stratum water at 30 min; the ratio of stratum water to gas does not change significantly from 30 to 40 min. The 15 min time point in the microscopic displacement experiment corresponds to Figure 5a and Figure 6a, and the 40 min time point in the microscopic displacement experiment corresponds to Figure 5b and Figure 6b.

The 40–70 min point in the microscopic displacement experiment marks the process of the drilling fluid displacement of stratum fluids. When drilling fluid starts to displace stratum fluid, the initial intrusion rate of drilling fluid is very fast, close to the intrusion rate of stratum water in the saturated stratum water stage of the microscopic model (the slope of the blue line from 0 to 30 min is close to the slope of the red line from 40 to 50 min), but after encountering small pores and narrow throats (at 50 min), there is no obvious fluid flow during the following 50 to 70 min, and the microscopic model reaches a fully damaged state. The 50 min time point in the microscopic displacement experiment corresponds to Figure 5c and Figure 6c, and the 70 min time point in the microscopic displacement experiment corresponds to Figure 5d and Figure 6d.

**Figure 8:**
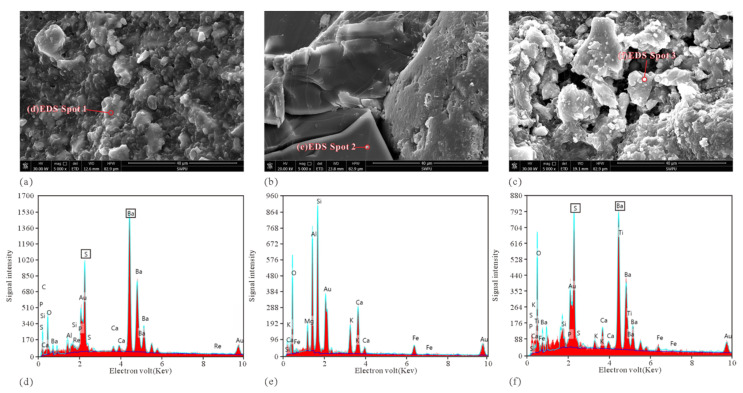
SEM image and energy spectrum analysis results: (**a**) SEM image of solid-phase composition of drilling fluid; (**b**) SEM image of the core sample before damage; (**c**) SEM image of the core sample after damage; (**d**) energy spectrum analysis result for solid composition of drilling fluid; (**e**) Energy spectrum analysis result of core sample before damage; (**f**) Energy spectrum analysis result of the rock sample after damage.

The photos of the four core plugs damaged by drilling fluid through core flooding experiments are shown in Figure 7c. The core plugs after core flooding experiments have obvious characteristics of drilling fluid damage. The permeability measurements of the four core plugs before and after damage by drilling fluid are shown in Figure 7b. The permeability of the four cores shows a significant decrease after damage by drilling fluid. The permeability decline ratios (Equation (2)) of the four core plugs are 48.27%, 50.10%, 30.23%, and 48.12%, respectively, indicating that the drilling fluid did damage the reservoir in the study area. The results of SEM photo and energy spectrum analysis of the four core plugs before and after damage by drilling fluid are shown in Figure 8. The diameter of the solid particles in the drying drilling fluid ranges from 0.1 to 30.0 μm, and the characteristic elements indicated by the solid-particle energy spectrum analysis are sulfur (S) and barium (Ba) (corresponding to the BaSO_4_ particles in the drilling fluid, Figure 8a,d). The SEM photos of the core samples before damage by drilling fluid show that the grain surface in the undamaged rock was very clean with very few debris particles. The energy spectrum analysis indicates that the main characteristic element of the grains in the undamaged rock is silicon (Si) (corresponding to SiO_2_ grains in the rock, Figure 8b,e). The SEM photos of the core samples damaged by drilling fluid show that there are a large number of clastic particles on the surface of the grains of the damaged rock (Figure 8c), and the characteristic elements indicated by the energy spectrum analysis of these clastic particles are sulfur (S) and barium (Ba) (Figure 8f), which are the same as the characteristic elements of solid particles in drilling fluid, and the size and morphology of both particles are also similar. Therefore, it can be speculated that the large number of clastic particles appearing on the surface of the damaged core samples came from the BaSO_4_ particles in the drilling fluid.

Combining the results of MEM displacement experiments, core flooding experiments, permeability measurements and SEM observations, it is shown that the main forms of damage of the low-porosity and low-permeability sandstone in the research area by drilling fluid include solid damage and liquid damage: (1) solid particles (especially BaSO_4_) in drilling fluid ranging in diameter from 0.1 to 30.0 μm move with the drilling fluid to the small pores and narrow throats, blocking the pores and throats and causing solid damage in the reservoir; (2) the white oil and water in the drilling fluid come into contact with the stratum water and natural gas (or crude oil) in the formation, forming an oil–water two-phase interface, a gas–water two-phase interface or an oil–gas–water three-phase interface, and these interfaces reach small pores and narrow throats in rock with the movement of the drilling fluid, generating a very large seepage resistance, forming water lock and hydrocarbon lock effects, and causing liquid damage. In the process of oil and gas production, the solid damage and liquid damage caused by drilling fluid intrusion into the reservoir significantly reduce the efficiency of oil and gas recovery.

## 5. Conclusions

In this study, a new MEM, along with displacement equipment, was designed. This equipment makes taking observations of the pore-scale drilling fluid displacement and researching of the liquid damage of drilling fluid possible. The MEM uses laser etching of a thin aluminum sheet to represent the rock pore network, and two thick glass sheets to hold the etched thin aluminum sheet and encapsulate it with a rubber sleeve. The fabrication and encapsulation of this MEM allow it to withstand higher confining pressure and displacement pressure and to conduct drilling fluid displacement experiments under higher pressure conditions. The major component of the displacement device is a high-pressure sleeve cavity with a transparent glass observation window, which allows the mutual displacement of gas, stratum water, and drilling fluid to simulate the process of formation damage by drilling fluid.

For the low-porosity and low-permeability sandstone reservoir of the Enping Formation in the Baiyun Sag of the Pearl River Mouth Basin, MEM-based displacement experiments, core flooding experiments, permeability measurements, and SEM observations before and after damage by drilling fluid were carried out. The results show that the main forms of low-porosity and low-permeability sandstone damage by drilling fluid include solid damage and liquid damage; solid particles (especially BaSO_4_) in drilling fluid move to the small pores and narrow throats in the rock and then block them; the white oil and water in the drilling fluid mix with the stratum fluid, forming an oil–water two-phase interface, gas–water two-phase interface or oil–gas–water three-phase interface, causing water lock and hydrocarbon lock effects and leading to liquid damage. Therefore, from the perspective of low-porosity and low-permeability reservoir protection and efficient oil and gas development, it is necessary to adjust the particle size of solid components in the drilling fluid, in addition to optimizing the material and ratio of liquid components in the drilling fluid.

## Figures and Tables

**Table 1:** Composition of the oil-based drilling fluid.

Composition	Concentration (kg/m^3^)
Water	0.12
Base oil	0.5
Calcium chloride	43.6
Amorphous silica	5.0
Silica gel	3.5
Lime	42.8
Amines and tallow alkyl	30.0
Organic minerals	14.3
Barite	786.0
Poly(oxy-1,2-ethanediyl),.alpha.-(carboxymethyl)-.omega.-(9-octadecenyloxy)-,(Z)-	11.5
Fatty acids, tall-oil, reaction products with diethylenetriamine, maleic anhydride, tetraethylenepentamine, and triethylenetetramine	31.4
Fatty acids, tall-oil, reaction products with amines, maleic anhydride, distillates, hydrotreated light, kerosine-unspecified, and rosin	17.2
Fatty acids, tall-oil, reaction products with diethylenetriamine, maleic anhydride, tetraethylenepentamine, and triethylenetetramine	5.8
